# Preconception counselling initiated by general practitioners in the Netherlands: reaching couples contemplating pregnancy [ISRCTN53942912]

**DOI:** 10.1186/1471-2296-7-41

**Published:** 2006-07-07

**Authors:** J Elsinga, KM van der Pal-de Bruin, S le Cessie, LC de Jong-Potjer, SP Verloove-Vanhorick, WJJ Assendelft

**Affiliations:** 1 Department Public Health and Primary Care, Leiden University Medical Center, Leiden, The Netherlands; 2 TNO Quality of Life, Department of Child Health, Prevention and Physical Activity, Leiden, The Netherlands; 3 Department of Medical Statistics, Leiden University Medical Center, Leiden, The Netherlands; 4 Department of Paediatrics, Leiden University Medical Center, Leiden, The Netherlands

## Abstract

**Background:**

To maximise the potential for reducing the risk of adverse pregnancy outcomes, preconception counselling (PCC) is used to inform couples contemplating pregnancy about general and personal risk factors. Many initiatives have been developed to provide PCC, but none offers it routinely in a presumed low-risk population.

The objective of the study was to investigate the extent to which women contemplating pregnancy can be reached when a PCC programme is routinely offered by general practitioners (GPs).

**Methods:**

30 GPs actively offered PCC to all women aged 18 to 40 over a three-year period. GPs reviewed lists of these women and excluded women with adverse social circumstances. The remaining women received an invitation for PCC. They were requested to indicate whether they were interested in PCC, and if so, when they were contemplating pregnancy. Those both interested and contemplating pregnancy within one year were invited for PCC. All pregnancies occurring within one year of an invitation were monitored. Response rates and percentages of pregnancies preceded by an invitation or actual attendance to PCC were calculated.

**Results:**

Overall, 72–75% of the interested responders, who returned the risk-assessment questionnaire (80%), actually attended PCC. However, the GPs excluded a large number of women. In 2002 27% of all pregnancies occurred in the group of women who had been interested and had indicated that they hoped to get pregnant within one year. Another 33% of the pregnancies occurred in the group of women who had been excluded, 13% in the group who had not responded, and 14% in the group who had not been interested.

**Conclusion:**

A quarter of the women who became pregnant in the year after the invitation were reached in time. In order to increase this number, methods should be developed to decrease the exclusion of women by the GPs and to increase women's response.

## Background

At least 20% of the clinically recognised pregnancies in the Western world have an adverse outcome, such as miscarriage, stillbirth, preterm birth, low birth weight or a congenital abnormality. [1,2]. The risk of such an outcome is known to be increased by risk factors such as smoking during pregnancy and non-compliance with folic acid use. In the development of the embryo, the 3^rd ^to 8^th ^postconceptional weeks of pregnancy are the most sensitive. To optimise maternal health before pregnancy and to make maximum use of the potential for reducing the risk of an adverse outcome, women should be offered information on risk factors – and thus take preventive measures as early as possible, preferably before pregnancy [3-10].

Different ways of offering preconception counselling (PCC) have been introduced to reduce adverse pregnancy outcomes. Moos et al. have described the integration of PCC within a regional primary care family planning clinic in the United States [11]; in China and Hungary an obligatory preconception counselling system was introduced [3,12]; and Jack et al., by describing the effects of PCC provided to women after a negative pregnancy test, have drawn attention to the many risk factors for a future pregnancy that are often present [13].

In the Netherlands, PCC has been offered to women known to be at a higher risk of an adverse pregnancy outcome [14]. PCC has also been provided by a clinical geneticist and an obstetrician after advertisement in local newspapers [15]. In no case, however, has it routinely been offered to all women with a potential desire to become pregnant, despite the fact that most adverse pregnancy outcomes occur in women in whom no increased risk has been identified.

The Netherlands provides an ideal setting for investigating the implementation of a PCC programme routinely offered by GPs: nearly all inhabitants are registered at a general practice, and a high percentage of pregnancies are planned (~80%) [16]. For this reason, the PCC project 'Parents to be' was established, in which all women aged 18 to 40 were offered PCC by their own GP [17].

To identify possible risk factors with regard to a future pregnancy, a questionnaire was sent to women who were interested in PCC and had indicated contemplating pregnancy within one year. When the questionnaire had been returned, the general practitioner invited the women and their partners for PCC. On the basis of the information provided by the questionnaire, the couples were given information on general and personal risk factors for adverse pregnancy outcome.

This new method of providing routine, systematic preconception counselling was introduced by 30 general practitioners in the western Netherlands. This article presents the results of this implementation, and quantifies the extent to which the programme was able to reach women contemplating pregnancy.

## Methods

The study "Parents to Be" was conducted as a randomised controlled trial in the Netherlands in order to investigate whether PCC would reduce adverse pregnancy outcomes. Sixty-seven general practitioners participated, mainly in the province of Zuid Holland (in the western Netherlands). All the GPs used the same electronic patient record (Medicom, Pharmapartners). The general practitioners were matched on the basis of the characteristics both of the general practitioner and of the practice and they consented to randomisation. Mostly pairs were formed, but sometimes clusters were larger due to the location of the general practice. Within each cluster general practices were randomised to either the intervention or control group. After randomisation, the intervention group consisted of 30 GPs and the control group of 37. The difference in number of GPs was due to a larger extent of GP's working part-time in the control group.

The mean total population size of the general practices in the intervention group was 2,662, ranging from 1,234 to 4,300 patients. The mean number of patients in our target group – women aged 18 to 40 – was 515, ranging from 268 to 1,145. The large differences in population size lay mainly in the fact that the majority of the general practitioners worked in health centres (with 33% working in a single-doctor practice), and that not every GP worked fulltime. The percentage of ethnic minorities among patients in the attending practices varied from 1 to 35%.

The following procedure for PCC was implemented at the general practices in the intervention group.

(i) In each practice, all women aged 18–40 years were selected, as most pregnancies occur in this age group in the Netherlands. GPs reviewed the selected women and excluded them for the following reasons: completed family, uterus extirpation, sterilisation, insufficient knowledge of the Dutch language, sub-fertility or infertility, pregnancy, definitive social circumstances (such as mental retardation), temporary social circumstances (such as a recent divorce), and "other" (such as no longer being registered at the practice or not being thought to be sexually active).

(ii) Each woman included received a letter from her GP, informing her of the possibility of PCC, and inviting her to attend PCC with her partner. Each woman was asked to indicate if she was interested in receiving PCC, and, if so, at which point in time she was contemplating pregnancy.

(iii a) Women who had indicated they were interested in advice and wished to become pregnant within a year were sent a risk-assessment questionnaire they could return to their GP. This contained questions on lifestyle, medical history, medicine use and hereditary diseases. Part of the questionnaire had to be completed by the partner (future father). After completion, the questionnaire had to be returned to the GP in a postage-paid envelope.

(iii b) Women who were not interested in the advice were asked to state their reasons. The answers were categorised as follows: no partner, no desire to have children (yet/any longer), already pregnant, information perceived to be sufficient, and other.

(iv) Together with their partner, women who had returned the questionnaire on their medical history were invited for preconception counselling by their GP. In summary, counselling comprised information on the general risk factors as well as information about preventive measures that are relevant to anyone contemplating pregnancy (e.g. folic acid, alcohol, tobacco, medicines and nutrition). Furthermore, information on personal risk factors relevant to the woman and her partner (e.g. advice on current medicine use, work-related hazards, or consultations with a specialist, e.g. obstetrician or clinical geneticist) was provided.

This procedure was followed three times (in 2000, 2001, 2002). In the first year, the GPs reviewed all women between 18 and 40 years old. In the second year, the procedure was limited to new female patients aged 18 to 40, women who had turned 18 since the first year, non-responders from the first year, and responders who had shown an interest in the first year but had stated that they would be contemplating pregnancy between one and five years in the future.

In the third year, the GPs reviewed the new female patients aged 18 to 40, women who had turned 18 years since the **second **time, non-responders from the **second **year, responders who had shown an interest in the **second **year but had stated that they would be contemplating pregnancy in between one and five years; interested responders from the **first **year who had not specified a specific period in which they desired to get pregnant; and women who had been excluded in the first and second years for temporary social circumstances, assuming these circumstances might have changed in the mean time.

In the first year (2000), the questionnaires were sent out in two-month batches. In the other two years, they were sent by the GPs' receptionists immediately after the answer-form had been returned.

In the first year the appointment for PCC was made by the doctors' receptionists within six weeks of their receiving the completed questionnaires; in the other years the women who received the questionnaire were requested to schedule the appointment for PCC themselves. These changes were made so as to avoid situations in which women became pregnant before they received the questionnaire or PCC, as had happened with some women during the first year in which PCC was offered.

As the response to and interest in new healthcare projects such as PCC always seems to follow an S-shaped curve – the response and interest being limited at first, but increasing rapidly until it levelled off – the results of the third year of PCC (2002) probably give the best impression of the way that the response and interest will evolve if PCC were implemented [18].

The general practices in the control group provided standard care.

The Medical Ethics Committee at Leiden University Medical Center approved this study.

### Data collection method

As well as ensuring the cumulative registration of women aged 18 to 40, we monitored all pregnancies occurring in this age group in which the first day of the last menstruation had fallen between 1 April 2000 and 1 April 2003. Data were collected on age of mother, duration of pregnancy, pregnancy outcome, place of birth, method of delivery, birth weight, gender, Apgar scores and congenital malformations.

Other characteristics were obtained from two questionnaires, the risk-assessment questionnaire referred to above, and a questionnaire assessing knowledge of pregnancy-related risk factors and preventive measures, that was sent to women in the intervention group prior to the first invitation for PCC [19].

### Statistics

The response rate was assessed for each year. Below we focus mainly on the last year that PCC was offered, as this best represents the situation that would pertain after implementation.

Frequencies were calculated for demographic variables, reasons for exclusion and reasons for not being interested. Because one of the major issues was to assess the impact of the offer of PCC on women contemplating pregnancy, we also identified all the women who became pregnant within a year of each invitation. It was established whether they had received an invitation for PCC, and, if so, what their response to it had been.

## Results

Table 1 presents the results of three consecutive years of inviting women to attend PCC at general practices. In 2002, the third year that women were invited to attend PCC, 5,866 women aged 18 to 40 were eligible in the 37 practices. Of those who were offered PCC (n = 3,154), 56% (n = 1,769) responded. Fifty-four percent (n = 962) of these responders indicated their interest in attending PCC; of these, 19% were contemplating pregnancy within one year. Of all the eligible women (n = 5,866) 49% had an intermediate level of education and 32% had a high level of education according to the classification of education of Statistics Netherlands (Table 2) [20]. More than half the women indicated they had been pregnant before.

**Table 1:** Results over three consecutive years of the invitation by 30 general practices to provide PCC.

Category of respondents	Year PCC was offered		
	
	2000	2001	2002
Total population to be approached	13,048	8,312	5,866
Review of population by GP			
Women included	7,872 (60%)	3,889 (47%)	3,154 (54%)
Women excluded	5,176 (40%)	4,419 (53%)	2,711 (46%)
Response of women included			
Yes	3,311 (42%)	1,702 (44%)	1769 (56%)
No	4,561 (58%)	2,187 (56%)	1,385 (44%)
Interest in PCC of responders			
Yes	1,480 (45%)	781 (46%)	962 (54%)
No	1,831 (55%)	921 (54%)	807 (46%)
Intended to get pregnant of interested responders			
Within one year	333 (23%)	205 (26%)	187 (19%)
Between one and five years	480 (32%)	282 (36%)	333 (35%)
Term unknown	607 (41%)	267 (34%)	419 (44%)
No desire for children, or for more children	35 (2%)	21 (3%)	21 (2%)
Pregnant	25 (2%)	6 (1%)	2 (0%)
Risk-assessment questionnaire			
Sent	378	147	179
Returned	260 (69%)	87 (59%)	144 (80%)
Preconception counselling			
Women attending	176 (72%)	69 (73%)	103 (76%)
Women already pregnant before attending	52 (21%)	17 (18%)	18 (13%)
Women who had moved	4 (2%)	4 (4%)	0 (0%)
Women no longer interested	13 (5%)	3 (3%)	1 (1%)
Other	1 (0%)	2 (2%)	13 (10%)

**Table 2:** Demographic characteristics of the women eligible in the three years PCC was offered.

		2000	2001	2002
		n = 13,048	n = 8,312	n = 5,866
Age	< 20 years	568 (4%)	490 (6%)	491 (9%)
	20 to 24 years	2,084 (16%)	1,495 (18%)	1,431 (24%)
	25 to 29 years	2,735 (21%)	1,952 (24%)	1,494 (26%)
	30 to 34 years	3,250 (25%)	2,097 (25%)	1,371 (23%)
	> 34 years	4,410 (34%)	2,278 (27%)	1,079 (18%)
Marital status*	Married/cohabiting	2,903 (75%)	1,294 (76%)	607 (60%)
	Permanent relationship	351 (9%)	154 (9%)	174 (17%)
	Single	597 (16%)	264 (15%)	236 (23%)
Country of birth*	The Netherlands	3,593 (93%)	1,598 (93%)	949 (93%)
	Surinam/Netherlands Antilles	55 (1%)	25 (2%)	17 (2%)
	Turkey/Morocco	45 (1%)	15 (1%)	11 (1%)
	Other	152 (4%)	75 (4%)	41 (4%)
Education*	Basic	843 (23%)	384 (24%)	189 (19%)
	Intermediate	1,596 (44%)	691 (42%)	474 (49%)
	University/college	1,197 (33%)	550 (34%)	307 (32%)
Medical insurance*†	National health insurance	271 (68%)	124 (66%)	105 (63%)
	Insurance for civil service employees	13 (3%)	11 (6%)	9 (6%)
	Private insurance	114 (29%)	51 (27%)	51 (31%)
	Not insured	1 (0%)	2 (1%)	0 (0%)
Previous pregnancies*	Yes	1,701 (44%)	773 (45%)	644 (63%)
	No	2,177 (56%)	950 (55%)	377 (37%)

* Because these characteristics were collected from two different questionnaires that not all women received, the numbers do not add up to the total number of women per response group.

^† ^This characteristic was included in only one of the two questionnaires.

Due to repeated invitations to non-responders, 19% of the original non-responders responded of which 38% were interested in participating in the study in the following year. This response is included in the 56% overall response to the invitation.

During the first year, it became apparent that many women had already become pregnant, either before receiving the risk-assessment questionnaire, or before the actual appointment for PCC. In the first year in which the GPs offered PCC, 72% of the women who returned the questionnaire actually attended PCC. When the time between sending the questionnaire and scheduling the appointment for PCC was reduced, this percentage increased to 76% in 2002 (Table 1).

The majority of the women who were interested in PCC were aged between 20 and 34. There were fewer women with a basic education [20] among the responders than among women who did not respond to the offer, or who indicated that they were not interested (Table 3).

**Table 3:** Demographic characteristics per response-group in the first year PCC was offered.

2000		Excluded N = 5,176	Non responders n = 4,561	Not interested n = 1,831	Interested n = 1,480
Age	< 20 years	202 (4%)	193 (4%)	90 (5%)	83 (6%)
	20 to 24 years	632 (12%)	806 (18%)	268 (15%)	379 (26%)
	25 to 29 years	824 (16%)	1,081 (24%)	319 (17%)	510 (34%)
	30 to 34 years	1242 (24%)	1,128 (25%)	518 (28%)	362 (24%)
	> 34 years	2274 (44%)	1,354 (29%)	635 (35%)	146 (10%)
Marital status^‡^	Married/cohabiting	854 (76%)	1,124 (77%)	389 (73%)	537 (72%)
	Permanent relationship	101 (9%)	108 (7%)	49 (9%)	93 (13%)
	Single	173 (15%)	220 (15%)	96 (18%)	108 (15%)
Country of birth^‡^	The Netherlands	1,042 (93%)	1,353 (93%)	511 (95%)	688 (92%)
	Surinam/Netherlands Antilles	16 (1%)	24 (2%)	3 (1%)	12 (2%)
	Turkey/Morocco	21 (2%)	13 (1%)	3 (1%)	8 (1%)
	Other	42 (4%)	61 (4%)	15 (3%)	34 (5%)
Education^‡^	Basic	261 (25%)	366 (27%)	115 (23%)	101 (14%)
	Intermediate	470 (44%)	569 (41%)	208 (41%)	350 (50%)
	University/college	330 (31%)	437 (32%)	178 (36%)	252 (36%)
Medical insurance^‡§^	National health insurance	27 (69%)	45 (72%)	1 (50%)	198 (67%)
	Insurance for civil service employees	3 (8%)	3 (5%)	0 (0%)	7 (2%)
	Private insurance	9 (23%)	13 (21%)	1 (50%)	91 (31%)
	Not insured	0 (0%)	1 (2%)	0 (0%)	0 (0%)
Previous pregnancies^‡^	Yes	452 (40%)	570 (39%)	235 (44%)	444 (60%)
	No	689 (60%)	892 (61%)	299 (56%)	298 (40%)

^‡ ^Because these characteristics were collected from two different questionnaires only part of the women received, the numbers do not add up to the total amount of women per response group.

^§ ^This characteristic was included in only one of the two questionnaires.

The aim of inviting women to attend PCC is to reach women before they become pregnant. We investigated all pregnancies that occurred within a year of the invitation (Table 4). In 2002, the third year of our project, which we regarded as the most representative, 27% of the pregnancies occurred in the group of women who had been interested and had indicated that they hoped to get pregnant within one year. Another 33% was found in the group of women who had been excluded, 13% in the group who had not responded, and 14% in the group who had not been interested. Ten percent of the pregnancies occurred in the women who had been interested and who had indicated that they would contemplate pregnancy one to five years in the future. Although the pregnancies after the invitation in 2002 were monitored only for six months, the percentage of pregnancies that were preceded by PCC (20%) was almost three times higher than in the first year of PCC (8%).

**Table 4:** Pregnancies occurring within one year of an invitation to attend PCC.

Year of invitation	2000	2001	2002
Number of pregnancies	797	513	182
Review of population by GP			
Women included	567 (71%)	296 (58%)	122 (67%)
Women excluded	230 (29%)	217 (42%)	60 (33%)
Response of included women			
Yes	349 (44%)	168 (33%)	99 (54%)
No	218 (27%)	128 (25%)	23 (13%)
Interest in PCC of responders			
Yes	246 (31%)	119 (23%)	74 (40%)
No	103 (13%)	49 (10%)	25 (14%)
Desire to become pregnant of interested responders			
Within one year	166 (21%)	81 (16%)	51 (27%)
Between one and five years	42 (5%)	23 (4%)	18 (10%)
Term unknown	35 (4%)	14 (3%)	5 (3%)
No desire for children, or for more children	0 (0%)	0 (0%)	0 (0%)
Pregnant	3 (1%)	1 (0%)	0 (0%)
Attended PCC before pregnancy	60 (8%)	48 (9%)	36 (20%)

^||^Because data were not collected over the total year in 2002, the reported number of pregnancies within that year was less than in the other years.

Many pregnancies that occurred within one year concerned women who had been excluded from PCC. Table 5 shows the reasons given for exclusion in 2000, as well as those for the women who became pregnant within one year of the invitation of PCC in 2000. Many women were excluded due to social circumstances; a number had also been excluded on the grounds of "completed family". Most frequently mentioned reasons for non-response among those who became pregnant within one year after the offer were 'thought to be well informed' (36%) and no current child wish (20%).

**Table 5:** Reasons women were excluded by GPs in 2000, and reasons women who were invited to attend PCC in 2000 were not interested in PCC, both correlated with the pregnancies occurring in these groups within one year.

	Number of women	Pregnancies within one year
Reasons for exclusion^¶^		
Completed family	357 (23%)	2 (15%)
Uterus extirpation	3 (0%)	0 (0%)
Sterilisation (either women or men)	16 (1%)	0 (0%)
Not speaking Dutch	313 (20%)	2 (15%)
Sub-fertility or infertility	27 (2%)	1 (8%)
Pregnant	24 (2%)	1 (8%)
Definitive social circumstances	172 (11%)	3 (23%)
Temporary social circumstances	288 (19%)	3 (23%)
Not (sexually) active	328 (21%)	0 (0%)
Earlier participation in PCC	7 (1%)	0 (0%)
Other	1 (0%)	1 (8%)
Reasons for not being interested		
No partner	20 (3%)	0 (0%)
No current desire for children, or no desire for more children	553 (67%)	5 (20%)
Pregnant	32 (4%)	3 (12%)
Already well informed	86 (10%)	9 (36%)
Moved	15 (2%)	0 (0%)
Infertile (intentionally or unintentionally)	7 (1%)	0 (0%)
Other	38 (5%)	3 (12%)
No reason given	63 (8%)	5 (20%)

^¶ ^As the general practitioner did not always indicate a reason for exclusion, this is often missing.

## Discussion

In the last year, GPs who had routinely offered a PCC programme over three years excluded 45% of the women aged 18 to 40. Of the women who were approached, 56% responded to the invitation, 54% of them by indicating that they would be interested in PCC when contemplating pregnancy. In the same year, 20% of the pregnancies that occurred within one year of an invitation had been preceded by PCC. Participation by the population originally eligible increased after multiple invitations.

In each of the three years, a large proportion of the women aged between 18 and 40 were excluded by the GP. Within 12 months, a considerable proportion of these women had become pregnant. It thus appears that something in the relationship between GPs and their patients may interfere with a GP's ability to assess which women aged between 18 and 40 should be invited for PCC. GPs could be trained, given strict instructions or given limited reasons for exclusion to prevent this large-scale exclusion. If the invitation for PCC were sent by another authority, such as a local health authority, exclusion would also be more limited. In this way, more women would receive an invitation for PCC, thereby increasing the response and the number of women who received PCC on time. The lower number of excluded women might also have beneficial effects in the long run, as a greater number of women who did not yet desire to get pregnant when they received the invitation to attend PCC would nonetheless become aware of the possibility of PCC.

The way we offered PCC can be compared with other screening programmes offered by the GP, for instance cervical and mammal cancer screening programmes. The response to these programmes was initially rather low. In the following years the response to these programmes has increased and levelled off after several years [18]. A similar pattern occurred at two points in our study. The response increased from 42% in the first year to 56% in the third year (Table 1); after the first year, 8% of pregnancies had been preceded by PCC, a figure that increased to 20% after the third year (Table 4). The percentages may increase further until the plateau is finally reached. As about 80% of pregnancies in the Netherlands are planned, the plateau would ideally level off at a maximum of 80% [16].

Many women who became pregnant within a year of an invitation did not fall into the group of women who had indicated that they were contemplating pregnancy within one year. About 27% of them had not responded to the offer at all, and had been sent a second invitation in the year after the initial non-response. Of the non-responders who did respond to this second invitation, 19% were interested in PCC.

In addition, 10% of the pregnancies occurring within one year after an invitation occurred in the group of women who had wished to get pregnant between one and five years in the future. This shows that wishes or intentions regarding pregnancy can change quickly. Repeated invitations at short intervals might help to increase the response. Scheduling appointments for PCC for women who are contemplating becoming pregnant in one to five years may also lead to an increase in the number of PCC sessions that are given before women become pregnant.

The Parents to Be study was initially started as a randomised controlled trial. The general practices providing standard care were situated largely in the same cities as the intervention practices. To prevent women of the control group becoming aware of the possibility of PCC, large-scale media attention such as advertising in local newspapers was not possible – which may have limited the response to and interest in the PCC programme.

In the third year that PCC was offered, 27% of all women actually contemplating pregnancy had received an invitation for PCC before they became pregnant. This is in accordance with similar studies in the area of health-care projects. In a Dutch study in which couples were offered carrier screening for cystic fibrosis, it was shown that about 20% of the couples were actually contemplated pregnancy [21]. When people in Maastricht, a main town in the southern Netherlands, were made aware of a preconception clinic through the local media, 106 couples attended it in the first year [15]. Czeizel described that when PCC was offered on an obligatory basis throughout Hungary, only 10% of the couples that wanted a child were reached [3]. This shows how difficult it is to reach women with pregnancy wish and that it will take a lot of effort to reach them.

The results of our study indicate four main ways in which one might increase the interest in and subsequent attendance to PCC of women who wish to get pregnant. First of all, GPs should become more aware that many women contemplating pregnancy might not be reached if too many of them are excluded. Therefore a method should be developed to minimize exclusion by the GP or bypass the GP by sending the invitation by another health authority. Secondly, because it was difficult for women to estimate when they wished to become pregnant, invitations for PCC should not be limited to those indicating that they intended to become pregnant within a year. Thirdly, as a large proportion of pregnancies occurred in the group of non-responders a year after an invitation had been sent, the reasons for non-response should be explored. Finally, familiarity with PCC should be raised through education at school and increased focus in the mass media. As 80% of the pregnancies in the Netherlands are planned, it must surely be possible to improve the response. Simultaneous efforts in these areas may contribute to the successful implementation of PCC in the Netherlands.

## Conclusion

A quarter of the women who became pregnant in the year after the invitation were reached in time. In order to increase this number methods should be developed to decrease the exclusion of women by the GPs and to increase women's response.

## Competing interests

The author(s) declare that they have no competing interests.

## Authors' contributions

LCJ and SPV were involved in the study design.

JE, LCJ enrolled participants and monitored data collection with KMP.

JE, KMP, and SC were involved in analysing and interpreting the data.

JE and KMP wrote the draft manuscript.

KMP, WJJA and SPV supervised the project.

All authors read and approved the final manuscript.

## Pre-publication history

The pre-publication history for this paper can be accessed here:


